# A randomized control trial of primary care-based management of type 2 diabetes by a pharmacist in Pakistan

**DOI:** 10.1186/s12913-019-4274-z

**Published:** 2019-06-24

**Authors:** Zaida Javaid, Unaiza Imtiaz, Imtiaz Khalid, Hamid Saeed, Rehana Qadir Khan, Muhammad Islam, Zikria Saleem, Muhammad Farhan Sohail, Zeeshan Danish, Farah Batool, Naveed Anwer

**Affiliations:** 1 Section of Clinical Pharmacy, University College of Pharmacy, University of the Punjab, Allama Iqbal Campus, Lahore, 54000 Pakistan; 2 Murad Clinic, Near Shalamar Hospital Mughalpura, Shalimar Link Road, Lahore, Pakistan; 3 Ripha Institute of Pharmacy, Township, Lahore, Pakistan; 4 Institute of Pharmacy, Lahore College for Women University, Lahore, Pakistan; 5 Saulat Institute of Pharmaceutical Sciences, Quaid –i- Azam University, Islamabad, Pakistan

**Keywords:** Lahore, T2DM, Pharmacist, Blood pressure, HbA1c, Pakistan, Pharmaceutical care, Glycemic control, Randomized control

## Abstract

**Background:**

The role of a pharmacist in primary health care settings of Pakistan is still obscure. Thus, we aimed to demonstrate the pharmacist-led improvements in glycemic, blood pressure and lipid controls in type 2 diabetes mellitus (T2DM) patients of Lahore, Pakistan.

**Methods:**

The first open label, randomized control trial conducted at a primary health care facility of Lahore, Pakistan by enrolling 244 uncontrolled type 2 diabetes (hemoglobin A1 c, (HbA1c); 10.85 ± 1.74) patients. The pharmacological intervention included identification of drug related problems, drug interactions, change in dose, frequency and therapy switches in collaboration with physician, while non-pharmacological intervention consisted of diet, lifestyle and behavior counseling. Outcome measures were glycemic (HbA1c), blood pressure and lipid controls.

**Results:**

In intra-group comparison, compared to control arm (**C**, *n* = 52), subjects in the intervention arm (**I**, *n* = 83) demonstrated significant differences in process outcome measures; baseline vs final, such as HbA1c (**C**; 10.3 ± 1.3 vs 9.7 ± 1.3, *p* <  0.001, **I**; 10.9 ± 1.7 vs 7.7 ± 0.9, *p* <  0.0001), systolic blood pressure (SBP) (**C**; 129.9 ± 13.9 vs 136 ± 7.1, *p* = 0.0001, **I**; 145 ± 20.4 vs 123.9 ± 9.9 mmHg, *p* <  0.0001), diastolic blood pressure (DBP) (**C**; + 4, *p* = 0.03, **I**; − 7 mmHg, *p* <  0.0001), cholesterol (**C**; 235.8 ± 57.7 vs 220.9 ± 53.2, *p* = 0.15, **I**; 224 ± 55.2 vs 153 ± 25.9 mg/dL, *p* < 0.0001), triglycerides (**C**; 213.2 ± 86.6 vs 172.4 ± 48.7, *p* = 0.001, **I**; 273 ± 119.4 vs 143 ± 31.6 mg/dL, *p* < 0.0001) and estimated glomerular filtration rate (eGFR) (**C**; 77.5 ± 18.6 vs 76 ± 14.2, *p* = 0.5, **I**; 69.4 ± 21.3 vs 93.8 ± 15.2 ml/min/1.73m^2^, *p* < 0.0001). Likewise, inter-group improvements were more significant in the subjects of intervention group at final follow up in comparison to control for various process outcome measures; HbA1c (*p* < 0.001), SBP (*p* < 0.0001), DBP (*p* = 0.02), cholesterol (*p* < 0.0001), triglycerides (*p* < 0.0001), SCr (*p* < 0.001), eGFR (*p* < 0.001). Moreover, both male and female subjects exhibited similar responses towards intervention with similar improvements in outcome measures.

**Conclusion:**

These data suggested that pharmacist intervention in collaboration with physician in primary health care settings may result in significant improvements in glycemic, blood pressure and lipid controls in Pakistani population.

**Trial registration:**

The trial was registered retrospectively with International Standard Registered Clinical/soCial sTudy Number (ISRCTN) registry on July 26, 2017 under nutritional, metabolic, endocrine category with assigned registration # *ISRCTN22657497* and can be assessed at 10.1186/ISRCTN22657497

**Electronic supplementary material:**

The online version of this article (10.1186/s12913-019-4274-z) contains supplementary material, which is available to authorized users.

## Background

According to 2017 global estimates by International Diabetes Federation (IDF), there are 451 million people with diabetes between 18 and 99 years of age and these numbers were projected to increase to 693 million by 2045 [22]. The crude prevalence of diabetes mellitus (DM) in Pakistan was 6.9% in 2017 that was expected to increase to 8.5% in adults (20-79 years), thus placing Pakistan 10th on IDF ranking based on number of adults with diabetes [28]. Regardless of the therapeutic headway, the management of diabetes to attain strict glycemic control on a long term basis remains complicated and ponderous, which in case of failure leads to inadvertently poor cardiovascular and microvascular outcomes [36, 51]. Pakistan’s healthcare system mainly addresses the acute illnesses while the chronic disease management is not fully taken care of by the health care system [3]. This aloofness could be attributable to several health system and patient related factors, such as limited healthcare human resource to tackle much needed educational demands of diabetic patients, overcrowded hospitals and clinics, lack of pharmaceutical care services under the supervision of a clinical pharmacist, greater emphasis on treating patients rather than disease and therapy related education and counseling, poverty/affordability, lack of education/poor health literacy, patient’s easy access to non-evidence based remedies practiced by hakeems (herbalist) and quacks - all leading towards the ineffective management and unwanted progression of diabetes [7, 46, 47].

According to World Health Organization (WHO)-Diabetes Country Profile 2016, Pakistan’s National response to diabetes completely lack comprehensive healthcare policies, guidelines and monitoring services for the disease. There are no operational policies and suggestible action plan in the health care system of Pakistan for an effective management of diabetes to control disease modifying risks, such as physical inactivity, weight gain and obesity [49]. Over and above there is complete absence of evidence based national diabetes standards or guidelines on patient’s education and treatment, tenuous referral criteria from primary to tertiary care and absence of diabetes registry and national risk factor surveys [49]. Additionally, in public health settings, there is a provision to obtain oral hypoglycemic agents (OHA), like Metformin and Sulfonylureas but insulin and related amenities are generally not available in primary healthcare facilities, e.g. blood glucose measurement, oral glucose tolerance test (OGTT), HbA1C test, dilated fundus examination, foot vibration perception by tuning fork, foot vascular status by doppler, urine strips for glucose and ketone measurement, and procedures like retinal photocoagulation, renal replacement therapy by dialysis or transplantation [49].

In many developed countries, pharmacist is considered as a pivotal member of a health care workforce and is the most accessible healthcare professional [35]. The role of a pharmacist as member of health care team has been evaluated in various randomized trials in community and clinical settings in managing and sustaining optimal glycemic, blood pressure and lipid controls to avert diabetic complications ([1];M. [5, 19, 20, 27, 33, 34, 38, 42, 44]).

According to 2015 trend report by the International Pharmaceutical Federation (FIP) on global pharmacy workforce intelligence, the median density of pharmacist in Pakistan stands at 0.51, 0.69 and 0.68 per 10,000 population in 2006, 2009 and 2012, respectively [15, 35] almost 12.7 times lower than the mean density of pharmacist in high income countries. Besides, Pakistan lacks doctor-patient-pharmacist loop, defunct pharmaceutical care plan for the management of T2DM with almost no operational policies and strategies to promote appropriate self-management/care practices to prevent disease related complications, disability limitation and encourage apropos rehabilitation, which the diabetic patients must learn to adopt during their visits to the clinics [13]. Currently, there are no published reports from Pakistan on the effectiveness of a standardized and structured model/algorithm for pharmacists to afford and deliver diabetes management services in any level of health care [12]. Therefore, it is high time to start engaging and empowering pharmacist in primary care settings of Pakistan to afford a thoughtful pharmaceutical care plan encompassing multifactorial pharmacological and non-pharmacological approaches that can be tailored according to the needs of individual patients with T2DM for a better glycemic, blood pressure and lipid controls to avert diabetes related complications in Pakistani population. Therefore, the current study is aimed at evaluating the pharmacist’s led improvements in glycemic, blood pressure and lipid controls in T2DM patients of Lahore, Pakistan.

## Methods

### Ethical approval

The study was approved by Ethical Committee of Human Research, Punjab University College of Pharmacy, University of the Punjab, Lahore, reference #; HEC/1000/PUCP/1926. The informed consent was obtained (Additional file 3) from all the subjects. The consent was also obtained from the participants to publish the study results.

### Study design

A prospective parallel, single centered, randomized control trial, “**S**tudy on **A**1c **M**anagement by **P**harmacist in **L**ahor**e** (SAMPLe) was conducted at a primary care clinic of Lahore, Pakistan. Data were collected over a period of 9 months with three follow ups in total, each follow up after every three months, as per American Diabetes Association, A. D [8] guidelines, which recommends to measure HbA1c after every three months to assess the glycemic control [11].

#### Sample size

Sample size was estimated based on the prevalence of diabetes in Pakistan [43], i-e., 6.9%, using Daniel formula [25], where the prevalence of diabetes in Pakistan is 6.9%, so *P* = 0.069, while *Z* = 1.96 (for 95% level of confidence) and d = 0.05.

$$ n=\frac{Z^2\ P\left(1-P\right)}{d^2} $$

The calculated samples size was found to be 99. However, the data of 150 patients, 150 in each arm, were collected to compensate for the missing or dropouts.

#### Study settings

The study was carried out at a primary care facility, Murad clinic Shalamar link road, Lahore, under the supervision of a general practitioner (Additional file 3). The clinical setup consisted of 3 physicians, 1 qualified dispenser, 1 coordinator, 1 patient facilitator, 1 lab technician and 1 pathologist. All patients first approached patients’ facilitator and later transferred to coordinator for consulting physician, which after consultation will contact the coordinator again to get medicine from dispenser and later to a pharmacist. The last part is only applicable for the patients of intervention arm for education and counseling.

#### Patient recruitment

Un-controlled T2DM patients (HbA1c > 8%) were provided with information on the trial conduct and operational procedures by the research pharmacist. The subjects were recruited from March 20, 2016 – August 20, 2016. The trial ended on June 03, 2017.

#### Randomization

**Fig. 1:**
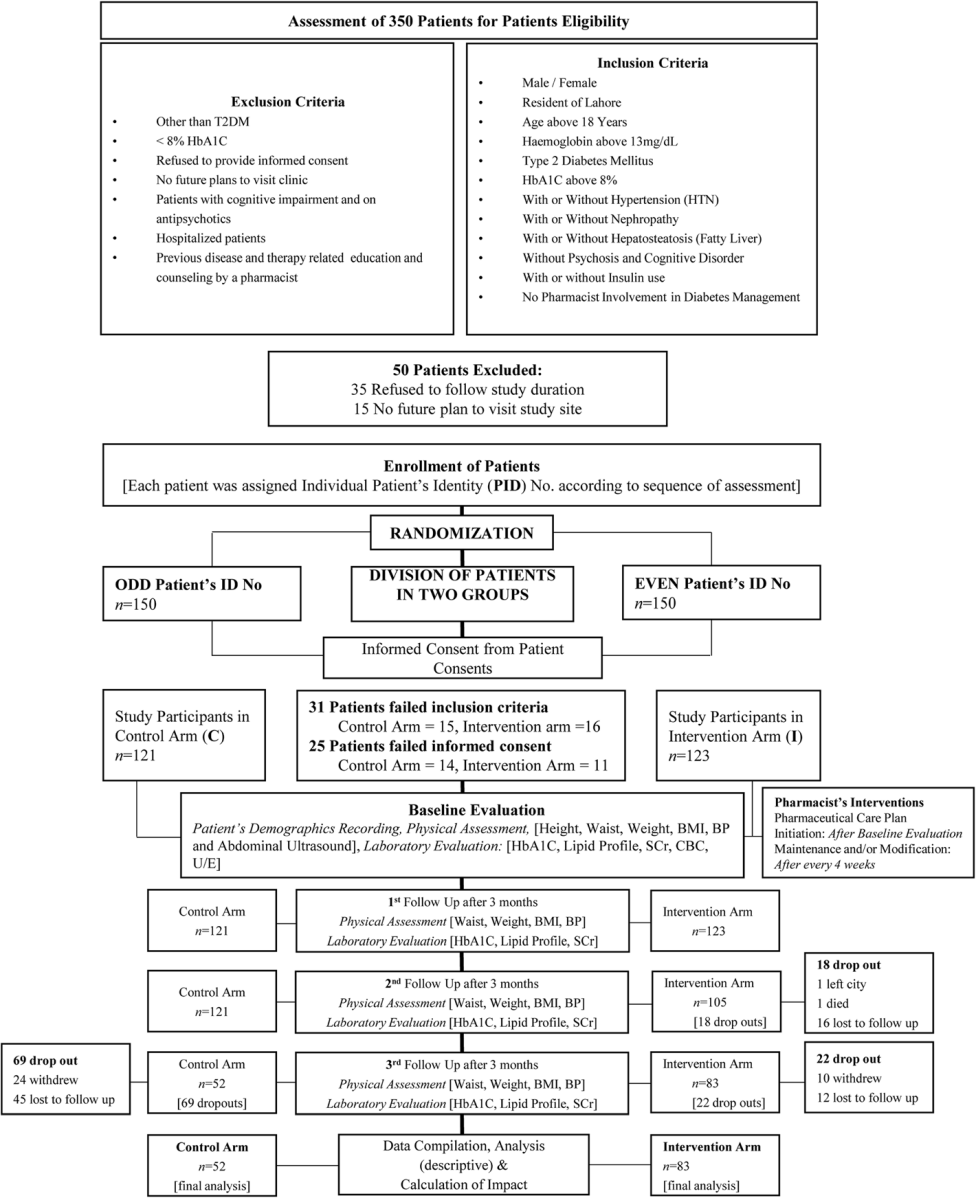
Description of the study participants and design

All eligible subjects were assigned patient’s identification numbers. Subjects with even numbers were segregated into intervention arm (*n* = 150), while odd number subjects into control arm (*n* = 150), followed by baseline evaluation (Fig. 1) by a pharmacist.

#### Baseline evaluation

Patient’s baseline evaluation, overall and gender specific, was done for the participants that included demographics, physical assessment parameters and laboratory measurements (Additional file 1: Table S1). This was followed by assessment of baseline clinical characteristics of the participants in the control and intervention arm (Additional file 1: Table S1).

#### Blinding

Open label with no blinding, both the pharmacist and physician knew about the subjects intervened.

#### Trial registration

*ISRCTN22657497 *(10.1186/ISRCTN22657497).

#### Missing data or dropouts

There were 40 drop outs in the intervention arm (12 withdrew and 28 lost to follow up) and 69 in the control arm - 24 withdrew and 45 lost to follow up. Thus, the final analysis was on 52 and 83 patients in the control and intervention arm, respectively (Fig. 1). The patients in control arm continued their participation till second follow. Afterwards 69 patients were dropped out (follow up failure) due to dissatisfaction towards disease management, failure to achieve treatment goals, switched to other physicians and lack of interest in the study.

### Study population

Out of total 300 eligible un-controlled T2DM patients enrolled in the study, 56 failed to provide the informed consent. Uncontrolled diabetes was confirmed by HbA1c levels above 8%, according to ADA guidelines [9]. Thus, only 244 subjects were considered for baseline evaluation (Fig. 1) as per study inclusion and exclusion criteria.

#### Inclusion criteria

Un-controlled T2DM patients, above 18 years of age, irrespective of gender, ethnicity and social class, must be visiting the clinic for the last 6 months, with or without concomitant disease and willing to participate in the study.

#### Exclusion criteria

Patients below 18 years of age, cognitive impairment, missing visits in the previous six months, cognitive impairment and not willing to participate were excluded.

### Research procedures

The base line evaluation of participants was performed using data collection form (Additional file 2). The initial education & counseling was about disease, therapy, lifestyle modifications, self-monitoring of blood glucose and regarding drug related problems. Patients were asked to visit every 4 weeks for the assessment of pharmacological and non-pharmacological needs, while the routine follow up was every three months. Patients in both arms were informed about upcoming visits through telephonic calls or short message service (sms). The findings were documented and assessed to formulate Individualized Pharmaceutical Care Plan.

#### Pharmaceutical care plan

A comprehensive pharmaceutical care plan for the patients of intervention arm was designed by the intervening pharmacist (Additional file 1: Figure S1). Briefly, pharmacist work up for drug therapy (PWDT) included CORE (Condition, Outcome, Regime, Evaluation) and PRIME (Problem, Risk, Interaction, Mismatch, Efficacy) components. CORE components included condition/needs, outcome, regimens and continuous evaluation. PRIME components included pharmaceutical based problems (non-adherence, monitoring & screening), risk to patients (Adverse Drug Reactions (ADRs), allergy), interactions, mismatch (drugs and patient’s needs) and efficacy parameters (Additional file 1: Figure S1). On every visit after 4 weeks, the intervening pharmacist assessed the patient’s individual needs for the modification of Pharmaceutical Care Plan, based on patient’s response towards intervention and self-monitoring record of blood glucose and blood pressure measurements. This was done in the form of progress notes followed by discussions with the study physician for treatment modification, if any.

At each follow up visit, every three months, the average pharmacist patient encounter time was between 15 and 30 min.

#### Physician collaboration

After baseline assessment and documentation of patient’s clinical and laboratory parameters, at each follow up, patients in the intervention arm were received by a pharmacist in a designated room for education and counseling. Patients were bound to bring their laboratory reports and daily blood glucose and blood pressure record, and any necessary documents. Thereafter, progress notes were made regarding patient’s pharmacological (drug related problems and suggestions for therapy changes) and non-pharmacological needs. Non-pharmacological needs were taken care by providing verbal and readable (leaflets, instructions on the back of prescriptions) education and counseling sessions/materials, e.g., insulin administration, medication adherence issues and self-care and monitoring. Progressive revisions in pharmacological needs, drug related problems or intensification of existing therapy, at each follow up taking into account patient’s current medications, clinical and biochemical data were suggested in consultation with study physician on the same day, majorly by progress notes and after a separate discussion in physician’s room or rarely in the presence of a patient, if necessary. Patients were provided instructions on the prescription or on a separate page.

The patients in the control group continued treatment from their physicians and their laboratory reports were collected at each follow up by the physician and nurse as part of a routine checkup.

#### Patient-pharmacist interaction

Patient-pharmacist interaction occurred in a separate room designated for patient education and counseling enrolled in the intervention arm, i-e. right after the patient-physician encounter and pertinent laboratory tests. The average time of sessions was 15–30 min, but time varied depending upon the patient’s needs and issues. During this session pharmacist performed case evaluations, made progress notes and develop intervention plan addressed to the physician. A comprehensive pharmaceutical care plan was designed for each patient by identifying patient’s non-pharmacological and pharmacological needs. A detailed description of pharmacist’s intervention is given below in Pharmacist Intervention section.

### Description of Pharmacist’s intervention

The intervention arm patients were categorized into three groups namely A, B & C according to follow ups attended, as shown in Additional file 1: Figure S2. The pharmacist intervention was based on Diabetes Self-Management Education criteria (DSME) [30] described below.

#### Pharmacist work up for drug therapy (PWDT)

Pharmacist’s work up of drug therapy (PWDT) included documentation of patient’s demographics and patient medical information, such as medical, social, dietary, family and medication history followed by laboratory results and physical findings. After documentation, pharmacotherapy problems associated with the use of pharmaceuticals were assessed related to patient risk, drug interactions, non-adherence, therapy mismatches and efficacy (drug choice, dose, route and frequency). Pharmacotherapy plans were made based on patient’s medical and non-medical needs to determine most suitable therapeutic regimen, revisions of the existing and appropriateness of the new, and behavioral recommendations on diet, lifestyle modifications, self-monitoring of glucose levels and self-care.

#### Pharmacological interventions (DRPs)

After patient-physician encounter at each follow up, patients in the intervention arm were received by the pharmacist in a designated room for patient education and counseling. Pharmacotherapy follow up activities were focused on issues related to patient’s changing needs with regards to treatment effectiveness and safety. Thus, pharmacotherapy interventions (PI) were processed after careful evaluation of medical prescription considering drug choice/switches, dose, overdose, effectiveness, interactions and adverse drug reactions, via progress notes and case evaluation to establish an intervention plan addressed to the physician. Drug related problems (DRPs) like medicine timing, frequency, uncontrolled hyperglycemia and episodes of hypoglycemia were discussed with the physician. Drug interaction like concurrent use of insulin with pioglitazone, which increases the risk of edema, heart failure and hypoglycemia [21], was identified and reconciled by advising insulin and biguanide combination with evidence based discussion and physician’s consent. Following medication reconciliation, patients were advised on the access, storage and use of medicines.

#### Oral hypoglycemic agents and insulin administration

Patients were briefed about the time of administration, i-e; with meal or 15–30 min before meal, dosing schedule, possible side effects and frequently interacting drugs. Patient were counselled on insulin administration focusing on injection technique, use of syringe, insulin pen and correct ways of administration. Subjects were told about the best injection sites, i-e., thighs and abdomen, and remember to keep rotating the injection site.

#### Medication adherence and treatment goals

Patients were thoroughly probed by asking close ended questions, such as did you ever missed to take your dose?, at times, are you careless about taking your drugs? and if you feel better, do you stop taking medicine etc., to determine the reasons behind the skipped doses to later counsel them regarding the importance of timely doses in controlling the disease symptoms and to prevent disease related complications. Moreover, patients were also asked about appearance of any side effects or allergies.

Besides, patients were also briefed how to assess episodes of hypoglycemia and hyperglycemia and ways to avoid them.

Patients were also briefed about the goals of therapy that is necessary to achieve effective glycemic control, i-e. fasting plasma glucose levels of ≤110 mg/dL, random glucose of ≤180 mg/dL, while HbA1c levels should be < 7%, measured every 3 months. Blood pressure goals for diabetic patients were < 140/90 mmHg, yet, pharmacist intervention was initiated on BP of 130/85 mmHg. For lipid goals, low density lipoprotein (LDL) value of < 100 g/dL was set for diabetic patients, thus LDL value of > 130 mg/dL was considered cut off value for starting statins.

#### Dietary and lifestyle modifications

Dietary plans were tailored according to patient’s needs considering different factors like weight reduction, hypertension, chronic kidney disease (CKD), chronic liver disease (CLD) and socioeconomic factors, according to ADA Guidelines [10]. Patients were recommended to have 20–30 min of exercise daily, moderately intense and vigorous physical activity, depending upon his/her abilities to encourage weight loss for a healthy Body Mass Index (BMI). Patients were advised brisk walk for 25–35 min in morning and after dinner, and stretch exercises while sitting only for those who were unable to go out for a walk.

Patients were counselled to adopt and adhere to lifestyle and behavioral modifications, such as self-regulation of carbohydrate intake, physical activity and medication doses based on the results of blood glucose monitoring.

#### Self-monitoring of blood glucose (SMBG)

Generally, patients that require SMBG were educated and trained to acquire basic skills and knowledge to use home blood glucose monitor and to record the results in an organized way. As a routine practice, patients in the intervention arm were advised for self-monitoring of blood glucose, especially when on insulin therapy, in case of modifications in prescription of hypoglycemic agent(s) and modifications in diet and physical activity. Specifically, regular SMBG (2–4 times per day) was recommended for patients using multiple daily injections of insulin, acutely ill and on medications or with illness known to cause hyperglycemia. Increased frequency of SMBG (≥ 2 times a day and as required) was recommended for patients utilizing medications known to cause hypoglycemia (when experiencing symptoms of hypoglycemia), entering a new life experience, such as a new job or change in working hours, unusual routine like stress, not meeting glycemic targets and to learn the effects of various meals on blood glucose levels.

#### Assessment of diabetic complications and diabetic foot care

Patients in the intervention arm were assessed and advised screening for the followings diabetic complications;

Patients were advised fundus examination to rule out diabetic retinopathy. For diabetic nephropathy, patients were advised urine test for proteinuria and blood test for glomerular filtration rate. For diabetic neuropathy patients were screened for numbness or reduced ability to feel pain, tingling sensation, increased sensitivity to touch and loss of reflexes in consultation with the primary care physicians.

Diabetic foot care included the routine examination of feet for cuts, bruises, cracks, blisters and soars. Major counseling points were aimed at maintaining hygienic conditions, which included change of socks every day, wear comfortable shoes and clip your nail straight across. Patients were told to avoid following actions, cutting their own corns and calluses, avoid using Over The Counter (OTC)/non-prescription medicine for treating corns or warts, use of lotions between the toes, walking barefoot and applying heat to the feet.

#### Counselling for personal care and hygiene

Following points were covered under this section;

Advice on skin care included routine examination for the appearance of any unusual discoloration, wart, carbuncle, bruises, cuts etc. On every visit, patient’s feet were examined by visual inspection and with monofilament. Patients were also advised to daily wash/clean the feet, moisturizing and self-examination of feet using mirror or with the help of a family member. Patients were counseled for eye examination in case of changes in their vision, e.g., appearance of spots in vision. Patients were also advised for oral hygiene and tooth brushing/ cleaning after every meal and referrals to dentist for occurrence of dental caries.

### Outcome measures

The outcome measures examined to assess the impact of pharmacist intervention in the management of T2DM are described below as;

#### Primary outcome measures

HbA1c and plasma glucose levels, measured by taking plasma venous samples and sending them to Trust laboratory and Citilab and Research center, Jinnah Hospital, Lahore.

#### Secondary outcome measures

Blood pressure and lipid profiles, measured via sphygmomanometer and by sending samples to Citilab and Research center, Jinnah Hospital, Lahore.

All the laboratory measurements were performed by laboratory technician while the samples were collected by a trained nurse.

#### Funding

Partially funded by Punjab University College of Pharmacy, HS/PUCP/1926.

### Data analysis

Data was analyzed using Statistical Package for Social Sciences (IBM SPSS 20). Descriptive statistics was used to compare frequency distribution patterns of categorical variables. Baseline characteristics between control and intervention arm were compared using Pearson chi-square. All continuous and discrete variables were reported as mean and standard deviations from their respective means. The outcome measures, blood glucose levels, HbA1c, blood pressure and lipid profiles were measured at the same time with similar procedures for patients in both the arms. The averages of outcome variables were computed for baseline and for each follow ups in both the arms. Intra-group variations in the means of continuous and discrete variables were compared between baseline and final follow up. Likewise, inter group differences in means of outcome variables were compared between final follow up of control versus final follow up of intervention arm. The means of all the laboratory parameters were compared between control and intervention arm, baseline vs final, and final of control vs final of intervention, on Microsoft Excel, version 2010, using paired student’s t-test. To examine the effect of pharmacist’s intervention on outcome measures, over 9 months period, from baseline to each follow up, repeated-measures analysis of variance (ANOVA) was used. An alpha value equal to 0.05 or less will be considered statistically significant.

## Results

### Overall participants baseline demographics and clinical characteristics

The demographic data and clinical characteristics are summarized in Additional file 1: Table S1. Data suggested that average age of the participants was 50 **±** 9.2 years with T2DM duration of 6.8 **±** 5.4 years and mostly married. The clinical characteristics included uncontrolled diabetes (**HbA1c;** 10.8% ± 1.7), average BMI of 30.7Kg/m^2^ **±** 5.7, average systolic (SBP) and diastolic blood pressure (DBP) of 138.9 **±** 19.3 and 89.7 **±** 11.364 mmHg, respectively. Other included, lipid profiles [**Cholesterol;** 226.7 **±** 53.6 mg/dL, triglycerides **(TG,** 231.8 ± 105.3 mg/dL), high density lipoproetine-C (**HDL-C,** 48.3 **±** 15.6 mg/dL), low density lipoprotein-C (**LDL-C,** 132 **±** 50.9 mg/dL) and **VLDL-C,** 46.4 **±** 21.1 mg/dL] and elevated serum creatinine levels;1 **±** 0.3 mg/dL (Additional file 1: Table S1).

Gender wise differences existed in average baseline values for BMI (**M:** 29.3 **±** 4.4, **F:** 31.3 **±** 6.2, *p* = 0.004), cholesterol (**M:** 238.3 **±** 61.3, **F:** 220.7 **±** 48.4, *p* = 0.026), LDL-C (**M:** 143.39 **±** 58.4, **F:** 126.2 **±** 45.9, *p* = 0.013) and serum creatinine (**M:** 1.1 ± 0.4, **F:** 1.02 ± 0.3, *p* = 0.36) (Additional file 1: Table S1).

### Participants baseline characteristics; control vs intervention

Patients baseline clinical characteristics of both arms are summarized in Table 1. At baseline, control (**C**) vs intervention (**I**), significant differences were observed regarding duration of T2DM (**C;** 7.6 ± 5.4, **I;** 6.1 ± 5.3, *p* = 0.029), *SBP* (**C;** 133 ± 15.4, **I;** 145 ± 20.9, *p* = 0.0001), *DBP* (**C;** 85 ± 10.4, **I;** 94 ± 10.7, *p* = 0.0001), *triglycerides* (**C;** 191 ± 79.8, **I;** 272 ± 112.1, *p* = 0.0001), LDL-C (**C;** 145 ± 48.5, **I;** 119 ± 50.3, *p* = 0.0001), *VLDL-C* (**C;** 38 ± 15.9, **I;** 54 ± 22.4, *p* = 0.0001) and *serum creatinine (SCr)* (**C;** 1.0 ± 0.3, **I;** 1.1 ± 0.4, *p* = 0.007) (Table 1). No differences were noticed at baseline with regards to age, treatment choices, *HbA1c, cholesterol*, weight, *HDL-C* and hemoglobin (Table 1).

**Table 1:** Baseline clinical characteristics of participants; control & intervention

Clinical Characteristics of Participants in Two Study Arms (At Baseline) [Mean ± SD]			
Parameters	Control Arm, *n* = 121	Intervention Arm, *n* = 123	*p*–values
*Age (Yrs)*	50.4 ± 7.7	50.3 ± 10.5	0.89
*Age at diagnosis of DM(Yrs)*	42.8 ± 7.9	44.2 ± 9.8	0.24
*Duration of DM (Yrs)*	7.6 ± 5.4	6.1 ± 5.3	0.029*
*Haemoglobin (mg/dL)*	13.2 4 ± 1.1	13.9 ± 1.1	0.69
*Weight (Kg)*	78.3 ± 14.4	77.5 ± 17.6	0.72
*Waist (cm)*	110 ± 16.5	109 ± 16.5	0.86
*BMI (Kg/m* ^*2*^ *)*	30.6 ± 4.9	30.8 ± 6.4	0.79
*Systolic BP (mmHg)*	133 ± 15.4	145 ± 20.9	0.0001**
*Diastolic BP (mmHg)*	85 ± 10.4	94 ± 10.7	0.0001**
*eABG (mg/dL)*	261 ± 49.8	268 ± 50.2	0.25
*HbA1c*	10.7 ± 1.7	11.0 ± 1.7	0.25
*Cholesterol (mg/dL)*	231 ± 55.7	223 ± 51.3	0.26
*Triglycerides(mg/dL)*	191 ± 79.7	272 ± 112.1	0.0001**
*HDL-C (mg/dL)*	48 ± 12.5	49 ± 18.2	0.45
*LDL-C (mg/dL)*	145 ± 48.5	119 ± 50.3	0.0001**
*VLDL-C (mg/dL)*	38 ± 15.9	54 ± 22.4	0.0001**
*Serum Creatinine (mg/dL)*	1.0 ± 0.3	1.1 ± 0.4	0.007*
Treatments			
*None*	2 (1.7%)	1 (0.8%)	
*OHA*	66 (54.5%)	72 (58.5%)	0.51
*OHA + Insulin*	43 (35.5%)	45 (36.6%)	
*Insulin*	10 (8.3%)	5 (4.1%)	

Abbreviation: *SD* Standard Deviation, *DM* Diabetes Mellitus, *BMI* Body Mass Index, *eABG* Estimated Average Blood Glucose, *HbA1c* Glycosylated Haemoglobin, *HDL-C* High Density Lipoprotein-Cholesterol, *LDL-C* Low Density Lipoprotein-Cholesterol, *VLDL-C* Very Low Density Lipoprotein-Cholesterol, *OHA* Oral Hypoglycaemic Agents, *I* Intervention, *C* Control, *M* Male, *F* Female

p-values; *p < 0.05–0.002, **p < 0.002–0.0001

### Intra and inter-group comparisons of process outcome measures

In intra-group comparisons, from baseline (**B**) to 1st, 2nd and final follow up (**F**), significant differences were observed in process outcome measures in the intervention arm (**I**), starting from 1st follow up till final follow up. Notables ones included, *HbA1c* (**B;** 11 ± 1.7, **1st;** 9.5 ± 1.6, **2nd;** 8.4 ± 1.1, **3rd;** 7.7 ± 0.9, *p* = 0.0001), *Systolic blood pressure (SBP)* (**B;** 145 ± 20.9, **1st;** 133 ± 14.2, **2nd;** 127 ± 10.6, **3rd;** 124 ± 9.9, *p* < 0.0001), *cholesterol* (**B;** 223 ± 51.3, **1st;** 187 ± 36.6, **2nd;** 169 ± 28.1, **3rd;** 153 ± 25.9, *p* < 0.0001), *Triglycerides (TG)* (**B;** 272 ± 112.1, **1st;** 195 ± 53.9, **2nd;** 164 ± 39.9, **3rd;** 143 ± 31.6, *p* < 0.0001), and *Serum creatinine (SCr)* (**B;** 1.1 ± 0.4, **1st;** 0.9 ± 0.2, **2nd;** 0.9 ± 0.2, **3rd;** 0.8 ± 0.1, *p* < 0.0001), while *estimated glomerular filteration rate (eGFR)* (**B;** 70 ± 2, **1st;** 80 ± 18.6, **2nd;** 87 ± 18.3, **3rd;** 94 ± 15.2, *p* < 0.0001) exhibited a significant increase (Table 2). While in the control group only *HbA1c* (**B;** 10.7 ± 1.7, **1st;** 10.6 ± 2.1, **2nd;** 10.2 ± 1.9, **3rd;** 9.7 ± 1.3, *p* = 0.001) and *Diastolic blood pressure (DBP)* (**B;** 85 ± 10.4, **1st;** 85 ± 7.9, **2nd;** 86 ± 9.8, **3rd;** 89 ± 4.2, *p* = 0.03) exhibited significant differences among baseline and the follow-ups (Table 2). However, the differences were similar in both the arms, baseline to final follow ups, for *LDL-C* and *VLDL-C* levels (Table 2).

**Table 2:** Inter and intra-group changes in process outcome measures in control and intervention arms

	Process Outcome Measures Intra-group comparison; baseline vs follow-ups Inter-group comparison; final control vs final intervention [Mean ± SD]										F_C_ vs F_I_ (*p*-values)
Process Outcomes	Control Arm					Intervention Arm					
	Baseline, *n* = 121	Follow up Every 3 Months			*p*-values	Baseline, *n* = 123	Follow up Every 3 Months			*p*-values	
		1st, *n* = 121	2nd, *n* = 121	3rd, *n* = 52			1st, *n* = 123	2nd, *n* = 105	3rd, *n* = 83		
Weight [Kg]	78.2 ± 14.4	78.4 ± 15.1	78.8 ± 14.9	76.3 ± 14.8	0.915	77.5 ± 17.6	75.9 ± 16.9	74.6 ± 16.2	73.7 ± 16.6	0.258	0.35
Waist [cm]	110 ± 16.6	109 ± 16.1	110 ± 16.2	106 ± 14.3	0.567	109 ± 16.5	107 ± 15.9	105 ± 15.3	103 ± 13.6	0.042*	0.28
BMI [Kg/m^2^]	30.6 ± 4.9	30.6 ± 5.1	30.8 ± 5.1	30.7 ± 5.6	0.986	30.7 ± 6.4	30.2 ± 6.2	29.4 ± 5.9	28.9 ± 5.9	0.131	0.06
HbA1c (%)	10.7 ± 1.7	10.6 ± 2.1	10.2 ± 1.9	9.7 ± 1.3	0.001**	11 ± 1.7	9.5 ± 1.6	8.4 ± 1.1	7.7 ± 0.9	0.0001**	0.0001**
eABG [mg/dL]	261 ± 49.8	257 ± 58.9	246 ± 54.7	232 ± 38	0.0036*	268 ± 50.2	227 ± 44.8	194 ± 32.1	174 ± 25.6	< 0.0001**	0.025*
SBP [mm/Hg]	133 ± 15.4	132 ± 13.4	134 ± 13.2	137 ± 7.1	0.082	145 ± 20.9	133 ± 14.2	127 ± 10.6	124 ± 9.9	< 0.0001**	0.0001**
DBP [mm/Hg]	85 ± 10.4	85 ± 7.9	86 ± 9.8	89 ± 4.2	0.03*	94 ± 10.7	89 ± 7.1	88 ± 6.1	87 ± 5.4	< 0.0001**	0.0001**
Cholesterol [mg/dL]	231 ± 55.7	229 ± 49.6	223 ± 43.7	221 ± 53.2	0.3	223 ± 51.3	187 ± 36.6	169 ± 28.1	153 ± 25.9	< 0.0001**	0.0001**
TG [mg/dL]	191 ± 79.8	186 ± 67.7	175 ± 53.1	172 ± 48.7	0.1	272 ± 112.1	195 ± 53.9	164 ± 39.9	143 ± 31.6	< 0.0001**	0.0001**
HDL-C [mg/dL]	48 ± 12.5	46 ± 10.9	47 ± 12.3	49 ± 13.9	0.437	49 ± 18.2	50 ± 11.4	49 ± 9.7	49 ± 7.9	0.975	0.794
LDL-C [mg/dL]	145 ± 48.5	146 ± 43.1	141 ± 39.4	82 ± 77.9	< 0.0001**	119 ± 50.3	99 ± 34.4	87 ± 26.1	76 ± 21.7	< 0.0001***	0.0001**
VLDL-C [mg/dL]	38 ± 15.9	37 ± 13.5	35 ± 10.6	20 ± 18.5	< 0.0001**	54 ± 22.4	39 ± 10.8	33 ± 8	29 ± 6.3	< 0.0001**	0.0001**
S Cr [mg/dL]	1.0 ± 0.3	1 ± 0.2	1.0 ± 0.2	1 ± 0.1	0.8	1.1 ± 0.4	0.9 ± 0.2	0.9 ± 0.2	0.8 ± 0.1	< 0.0001**	0.0001**
eGFR [ml/min/1.73m^2^]	77 ± 18.1	77 ± 15.8	76 ± 16.2	76 ± 14.2	0.5	70 ± 2	80 ± 18.6	87 ± 18.3	94 ± 15.2	< 0.001**	0.0001**

*Abbreviations: HbA1c* Glycated hemoglobin 1c, *eABG* Estimated average glucose, *SBP* Systolic blood pressure, *DBP* Diastolic blood, *HDL-C* High Density Lipoprotein-Cholesterol, *LDL-C* Low Density Lipoprotein-Cholesterol, *VLDL-C* Very Low Density Lipoprotein-Cholesterol, *Sr Cr* Serum Creatinine, *eGFR* Estimated Glomerular Filtration Rate, *F*_*C*_ Final control arm vs *F*_*I*_ final intervention arm

p-values; *p < 0.05–0.002, **p < 0.001–0.0001

Inter-group changes over time in the process outcome measures were compared between control final (**C**_**F**_) and intervention final (**I**_**F**_) follow ups as shown in the last column of Table 2. Data suggested that almost all the outcome measures exhibited significant differences at final follow ups between control and intervention arms, except for weight, waist, BMI and HDL-C (Table 2). Notable outcome measures demonstrating significant differences at final follow ups included *HbA1c* (**C**_**F**_**;** 9.7 ± 1.3, **I**_**F**_**;** 7.7 ± 0.9, *p* = 0.0001), *SBP* (**C**_**F**_**;** 137 ± 7.1, **I**_**F**_**;** 124 ± 9.9, *p* = 0.0001), *DBP* (**C**_**F**_**;** 89 ± 4.2, **I**_**F**_**;** 87 ± 5.4, *p* = 0.0001), *cholesterol* (**C**_**F**_**;** 221 ± 53.2, **I**_**F**_**;** 153 ± 25.9, *p* = 0.0001), *TG* (**C**_**F**_**;** 172 ± 48.7, **I**_**F**_**;** 143 ± 31.6, *p* = 0.0001), *SCr* (**C**_**F**_**;** 1 ± 0.1, **I**_**F**_**;** 0.8 ± 0.1, *p* = 0.0001) and *eGFR* (**C**_**F**_**;** 76 ± 14.2, **I**_**F**_**;** 94 ± 15.2, *p* = 0.0001) (Table 2).

### Gender wise comparison of process outcome Measures

Gender wise comparison of process outcome measures at final follow up for both control and intervention arms are summarized in Table 3. Data suggested that significant differences were observed in majority of the process outcome measures between final follow ups, control versus intervention arm, for male and female patients, such as *HbA1c* (**M;** 9.7 ± 1.4 vs 7.9 ± 0.7, *p* = 0.0001, **F;** 9.7 ± 1.3 vs 7.6 ± 0.9, *p* = 0.0001), *SBP* (**M;** 137.1 ± 6.4 vs 125.8 ± 8.3, *p* = 0.0001, **F;** 136.7 ± 7.6 vs 123.1 ± 10.5, *p* = 0.0001), *cholesterol* (**M;** 229.1 ± 78 vs 152.6 ± 27, *p* = 0.0001, **F;** 217 ± 36.5 vs 153.8 ± 25.6, *p* = 0.0001), *LDL-C* (**M;** 75.6 ± 93.5 vs 76.5 ± 25.2, *p* = 0.0001, **F;** 85.8 ± 67.3 vs 75.7 ± 20.2, *p* = 0.0001) and *Scr* (**M;** 0.9 ± 0.1 vs 0.8 ± 0.2, *p* = 0.0001, **F;** 0.9 ± 0.1 vs 0.8 ± 0.1, *p* = 0.0001). While, *VLDL-C* and triglycerides showed significant differences in female patients only, control versus intervention (Table 3).

**Table 3:** Gender wise comparison of process outcome measures at final follow up; control vs intervention

Parameters	Control Arm [Mean ± SD]		Intervention Arm [Mean ± SD]		Comparison of Male Participants	Comparison of Female Participants
	Final follow up, *n* = 52		Final follow up, *n* = 83			
	M = 17	F = 35	M = 26	F = 57	C_F_ vs I_F_ (*p*-values)	C_F_ vs I_F_ (*p*-values)
*HbA1c (%)*	9.7 ± 1.4	9.7 ± 1.3	7.9 ± 0.7	7.6 ± 0.9	0.0001**	0.0001**
*eABG [mg/dL]*	233.2 ± 39.9	231.4 ± 37.7	179.2 ± 21.6	171.4 ± 27.1	0.0001**	0.0001**
*SBP [mm/Hg]*	137.1 ± 6.4	136.7 ± 7.6	125.8 ± 8.3	123.1 ± 10.5	0.0001**	0.0001**
*DBP [mm/Hg]*	88.8 ± 3.8	88.7 ± 4.4	85.9 ± 4.7	87.1 ± 5.7	0.077	0.148
*Cholesterol [mg/dL]*	229.1 ± 78	217 ± 36.5	152.6 ± 27	153.8 ± 25.6	0.0001**	0.0001**
*TG [mg/dL]*	170.2 ± 47.3	173.4 ± 50	141.2 ± 24.1	143.1 ± 34.7	0.283	0.0001**
*HDL-C [mg/dL]*	48.3 ± 12.3	49.9 ± 14.8	47.8 ± 7.7	49.4 ± 8	0.210	0.170
*LDL-C [mg/dL]*	75.6 ± 93.5	85.8 ± 67.3	76.5 ± 25.2	75.7 ± 20.2	0.0001**	0.0001**
*VLDL-C [mg/dL]*	17 ± 18.5	22.3 ± 18.4	28.3 ± 4.8	28.6 ± 6.9	0.284	0.0002**
*SCr [mg/dL]*	0.9 ± 0.1	0.9 ± 0.1	0.8 ± 0.2	0.8 ± 0.1	0.0001**	0.0001**

*Abbreviation*: *SD* Standard deviation, *eABG* Estimated average blood glucose, *SBP* Systolic blood pressure, *DBP* Diastolic blood pressure, *TG* Triglycerides, *HbA1c* Glycated Haemoglobin, *HDL-C* High Density Lipid-Cholesterol, *LDL-C* Low Density Lipid-Cholesterol, *VLDL-C* Very Low Density Lipid-Cholesterol, *M* Male, *F* Female, *C*_*F*_ Control arm final, *I*_*F*_ intervention arm final

*p*-values; **p < 0.001–0.0001

### Impact on glycemic goals and other targets of diabetes care

As shown in Tables 4, 16.9% & 39.8% patients in the intervention arm achieved < 7% and < 8% HbA1c controls, respectively, compared to none (0%) and 5.8% in the control arm. Conversely, 69.2% in control arm sustained ≥9% HbA1c levels in comparison to 8.4% patients in the intervention arm (Table 4).

**Table 4:** Percentage of patients achieving glycemic and blood pressure goals

Outcome Measures	Control Arm				Intervention Arm			
	Follow Up				Follow Up			
	B, *n* = 121 (%)	1st, *n* = 121 (%)	2nd, *n* = 121 (%)	3rd, *n* = 52 (%)	B, *n* = 123 (%)	1st, *n* = 123 (%)	2nd, *n* = 105 (%)	3rd, *n* = 83 (%)
Glycaemic Goals								
**%** of patients achieving goal HbA1c **< 7%**	^a^0	2 (1.7)	2 (1.7)	0 (0)	^a^0	5 (4.1)	9 (8.6)	14 (16.9)
**%** of patients achieving goal HbA1c < 8%	^a^0	2 (1.7)	8 (6.6)	3 (5.8)	^a^0	14 (11.4)	28 (26.7)	33 (39.8)
**%** of patients achieving goal HbA1c < 9%	15 (12.4)	24 (19.8)	25 (20.7)	13 (25)	18 (14.6)	27 (22)	39 (37.1)	29 (34.9)
**%** of patients at HbA1c ≥ 9%	106 (87.6)	93 (76.9)	86 (71.1)	36 (69.2)	105 (85.4)	77 (62.6)	29 (27.6)	7 (8.4)
Blood Pressure Goals								
% of patients with SBP < 130 mmHg	42 (34.7)	41 (33.9)	27 (22.3)	4 (7.7)	22 (17.9)	31 (25.2)	39 (37.1)	45 (54.2)
% of patients with SBP ≥130 mmHg	79 (65.3)	80 (66.1)	94 (77.7)	48 (92.3)	101 (82.1)	92 (74.8)	65 (62.9)	38 (45.8)
% of patients with SBP ≥140 mmHg	49 (40.5)	40 (33.1)	45 (37.2)	29 (55.8)	84 (68.3)	51 (41.5)	19 (18.1)	8 (9.6)
% of patients with DBP < 80 mmHg	12 (9.9)	11 (9.1)	9 (7.4)	0 (0)	3 (2.4)	2 (1.6)	2 (1.9)	1 (1.2)
% of patients with DBP ≥80 mmHg	109 (90.1)	110 (90.9)	112 (92.6)	52 (100)	120 (97.6)	121 (98.4)	103 (98.1)	82 (98.8)
% of patients with DBP ≥90 mmHg	56 (46.3)	72 (59.5)	76 (62.8)	43 (82.7)	97 (78.9)	89 (72.4)	69 (65.7)	48 (57.8)

*Abbreviations: HbA1c* Glycosylated Haemoglobin, SBP Systolic blood pressure, *DBP* Diastolic blood pressure

^a^*Note:* Inclusion criteria of study was >8% HbA1c i.e. patients with uncontrolled diabetes mellitus

At final follow up, 7.7% patients in the control and 54.2% in the intervention arm achieved SBP goals of < 130 mmHg. While, 55.8% patients in the control and 9.6% in the intervention arm achieved SBP goal of ≥140 mmHg, respectively (Table 4). As for DBP, compared to 82.7% subjects in control arm, 57.8% subjects in the intervention arm achieved ≥90 mmHg (Table 4). In terms of cumulative reduction in HbA1c levels, combining 1st, 2nd & 3rd follow ups, the percentage of patients in control and intervention arms started to decline from 1 to 1.9% HbA1c reduction quartile onward, yet not a single patient in control arm met HbA1c reduction quartiles of 5–5.9% and onward, (Additional file 1: Table S2), however, a few in the intervention arm met HbA1c reduction quartiles of 6–6.9% (Additional file 1: Table S2).

## Discussion

The health care system of Pakistan lacks structured programs to manage non-pharmacological aspects of chronic diseases, such as therapy and disease related education and counseling, majorly due to lack of human resource and suboptimal health professionals to population ratio [41]. Despite clinical pharmacy education in Pakistan almost a decade ago, pharmacists in hospitals and primary care settings are providing conventional services [4, 14]. In the present study, pharmacist’s intervention regarding therapy, diet, lifestyle changes, self-care and management resulted in significant improvements in several process outcome measures, such as glycemic, blood pressure and lipid controls along with serum creatinine levels in comparison to control arm.

It has been reported that majority of adults with T2DM have at least one co-existing chronic condition [26], while almost 40% adult diabetic patients have 3 or even more, especially in patients having history of un-controlled diabetes [50]. Thus, the management of diabetes in patients with co-existing diseases require multifaceted approach by a team of health care professionals [12, 40]. Numerous literature evidences, randomized control trials (RCTs) in community and hospital settings, suggested that the addition of pharmacist to health care team for the management of chronic diseases, such as diabetes, not only resulted in strict glycemic control but also improved other targets of diabetes care, such as blood pressure and lipid controls [34, 45].

Data from the present study suggested that majority of the clinical parameters at baseline were similar, except for blood pressure, lipid profiles and serum creatinine. Nevertheless, except for LDL-C, mean values for blood pressure, VLDL-C, triglycerides and SCr were significantly higher in patents of intervention arm. However, these differences might not have a negative impact on trial primary outcomes regarding comparison between control and intervention arm, probably because of insignificant differences in HbA1c values between both the arms and higher mean baseline values of outcome measures in the intervention arm in comparison to control. Thus higher mean baseline values in intervention arm can only underestimate the pharmacist improvements in the outcome measures in comparison to control. Similarly, no significant differences were observed in baseline values in most of the outcome variables, especially HbA1c and blood pressure, between male and female patients, thus, the intervention footprint on these population cannot be attributed to gender base differences rather can be ascribed to pharmacist’s intervention in collaboration and cooperation of physician and patient, respectively.

In the present study, we observed a reduction in HbA1c of 3.3%, i-e., from 11 to 7.7% by introducing apt dietary and lifestyle modifications and allied self-management approaches by a pharmacist that presumably affected other targets of diabetes care. Conversely, in the control arm, there was only 1% reduction in HbA1c, from 10.7 to 9.7%, as a result contriving negligible effects on other targets of diabetes care, possibly due to still higher than ADA target goal of HbA1c levels. Numerous previous studies have suggested that pharmacist provision of pharmaceutical care in the management of diabetes could result in HbA1c reduction from 0.5–3.4% compared to almost no or minor changes in HbA1c levels in control subjects [6, 23, 24, 32, 37]. Our observation of 3.3% reduction in HbA1c - higher than already reported reductions, could be attributable to factors associated with Pakistan’s health care services and patient factors, i-e. lack of any form of disease or therapy related education & counseling for patients helpful in empowering them with the skills to self-manage their disease, poor health literacy and affordability among patients [46, 47] and lastly health seeking behavior of patients in Pakistan, i-e., most of the patients aspire less waiting time, spending more time with health care professional, free medicines, free laboratory tests, free health related education and counseling etc. [31]. Several randomized control trials (RCTs) demonstrated that pharmacist intervention resulted in greater number of patients achieving ADA target goal of < 7% HbA1c influenced by study duration and number of follow ups – for example, pharmacist intervention resulted in < 7% HbA1c levels in 23.4% patients in intervention arm compared to 15.2% in control arm at 6 months assessment [34]. Another study reported glycemic control in 45.4% subjects in the intervention arm and 30.3% in control arm at 12 months assessment [39]. Likewise, we observed that at final follow up greater number of patients (39.8%) in the intervention arm achieved < 8% HbA1c target compared to only 5.8% in the control arm, yet more surprisingly, not a single patient in the control arm achieved < 7% HbA1c target compared to 16.9% in the intervention arm. These findings clearly demonstrated that when pharmacist interventions appropriately addressed drug related problems (DRPs) [18] followed by pertinent lifestyle modifications [2, 17] and diabetes self-management education [16, 27], patient’s in the intervention arm exhibited greater reductions in HbA1c levels compared to control subjects.

Regarding impact on other targets of diabetes care, compared to control arm, starting from first follow up, i-e., after 3 months, the outcome measures were significantly improved at final follow up in the intervention arm in comparison to baseline, such as BMI, a switch from obese category (30.7 ± 6.4) to overweight category (28.9 ± 5.9), 21 mmHg reduction in SBP, 70 mg/dL reduction in cholesterol, 129 mg/dL reduction in triglycerides, 0.3 mg/dL reduction in serum creatinine and an increase of 24 ml/min/1.73m^2^ in eGFR. Similar to our findings, number of previously reported RCTs have suggested that pharmacist managed diabetes care could improve glycemic control and other outcome of diabetes care such body weight, blood pressure, LDL-C and cholesterol levels [29, 48]. According to a literature report, a blood pressure increase of 10 mmHg could increase the risk of cardiovascular events by 20% [1]. We found that compared to final follow up of the control arm, in the intervention arm, there was almost similar reductions in SBP in both males (12 mmHg) and females (13 mmHg) corroborating previous report that pharmacist intervention resulted in significant improvements in SBP and DBP [39]. Thus, it is reasonable to deduce that pharmacist intervention may contribute in averting the risk of cardiovascular events. Thus, in Pakistan, with almost negligible role of pharmacists in the management of chronic diseases (I. [4, 14]), this study signifies the pivotal role of a pharmacist in the management of diabetes in collaboration with primary care physician in a primary care settings.

The present study could have several practical implications in Pakistan’s health system, as it provides a first structured pharmaceutical care model in primary health care settings for the implementation of pharmaceutical care plan (PCP) in the management and care of T2DM patients by a pharmacist. Thus, a qualified pharmacist with prior knowledge of diabetes related pharmacological and non-pharmacological issues or a more formal training could suitably implement this model. Moreover, the current practice model utilizes and applies all the recommendations put forth by international associations and organizations for the management of diabetes using information system, evidence based management and multifaceted patient centered approaches in collaboration with a primary care physician. Moreover, this model initiates and encourages the development of good relationships between pharmacist, physician and patient, a factor that might have contributed in improved outcomes in patients of intervention arm.

Thus, followings are the recommendations to implement this practice model; the model can be implemented in all three tiers of health care system, i-e. primary, tertiary (hospitals) and even community care settings because of several reasons; this model allows frequent communication among physician, patient and diabetes management team, the model very well targets patients with poor glycemic control having multiple barriers to care, face to face interaction between pharmacist and the patient allows the patients to build a trusting relationship with mid-level provider, i-e. the pharmacist, which may improve self-care and adherence to medication and behavioral modifications, and finally, clinical pharmacist may have led to better management of complex therapy regimens. Similar practicing models can be developed and implemented for hypertension and dyslipidemia management.

### Study limitations

The study has a few limitations; though the enrolled subjects were from diverse backgrounds and localities of Lahore, it’s a single center study due to scarcity of funds and human resource. The study was of limited duration, therefore, long term impact of pharmacist’s intervention on disease outcomes could not be ascertained. Some of the baseline values of clinical variables, such as SBP, DBP, VLDL-C, triglycerides and serum creatinine were higher in intervention arm, which may indicate a possible underestimation of the impact of pharmacist’s intervention on these outcomes.

## Conclusion

In conclusion, results from the first RCT on pharmacist’s role in primary care settings in the management of T2DM in Lahore, Pakistan, employing individualized pharmaceutical care plan (PCP), demonstrated significant improvements in process outcome measures, such as blood glycemic levels, blood pressure, dyslipidemia, BMI and kidney functions.

## Additional files

Additional file 1: **Figure S1.** General Pharmaceutical Care Plan. **Figure S2.** Categorization of participants in intervention arm based on follow ups. **Table S1.** Patients Overall and Gender wise distribution of Baseline Demographics and Clinical Characteristics. **Table S2.** Percentage of Patients in HbA1c Reduction Quartiles. (DOCX 1053 kb)

Additional file 2: Data collection form. (DOCX 76 kb)

Additional file 3: Patient’s consent form. (DOCX 291 kb)

## Data Availability

Data will be available upon request to corresponding author at hamid.pharmacy@pu.edu.pk

## Abbreviations

B: Baseline
BMI: Body mass index
C: Cholesterol
C_F_: Final control
DBP: Diastolic blood pressure
DSME: Diabetes self-management education criteria
eABG: Estimated average blood glucose
eGFR: Estimate glomerular filtration rate
F: Female
F: Final follow up
HbA1c: Hemoglobin A1 c, glycated hemoglobin
HDL-C: High density lipoprotein - cholesterol
I: Intervention
I_F_: Final intervention
LDL-C: Low density lipoprotein - cholesterol
M: Male
OHA: Oral hypoglycemic agents
PWDT: Pharmacist work up of drug therapy
RCTs: Randomized control trial
SBP: Systolic blood pressure
SCr: Serum creatinine
SMBG: Self-monitoring of blood glucose levels
T2DM: Type 2 diabetes mellitus
TG: Triglyceride
VLDL-C: Very low density lipoprotein – cholesterol
