# Supplementary material for: A randomized control trial of primary care-based management of type 2 diabetes by a pharmacist in Pakistan
Source: BMC Health Serv Res. 2019 Jun 24;19:409. doi: 10.1186/s12913-019-4274-z (PMC6591859; doi:10.1186/s12913-019-4274-z)
Supplement: Supplementary file 1 — Figure S1. General Pharmaceutical Care Plan. Figure S2. Categorization of participants in intervention arm based on follow ups. Table S1. Patients Overall and Gender wise distribution of Baseline Demographics and Clinical Characteristics. Table S2. Percentage of Patients in HbA1c Reduction Quartiles. (DOCX 1053 kb) [file 12913_2019_4274_MOESM1_ESM.docx]

**Additional file 1**

**Figure S1.** General Pharmaceutical Care Plan


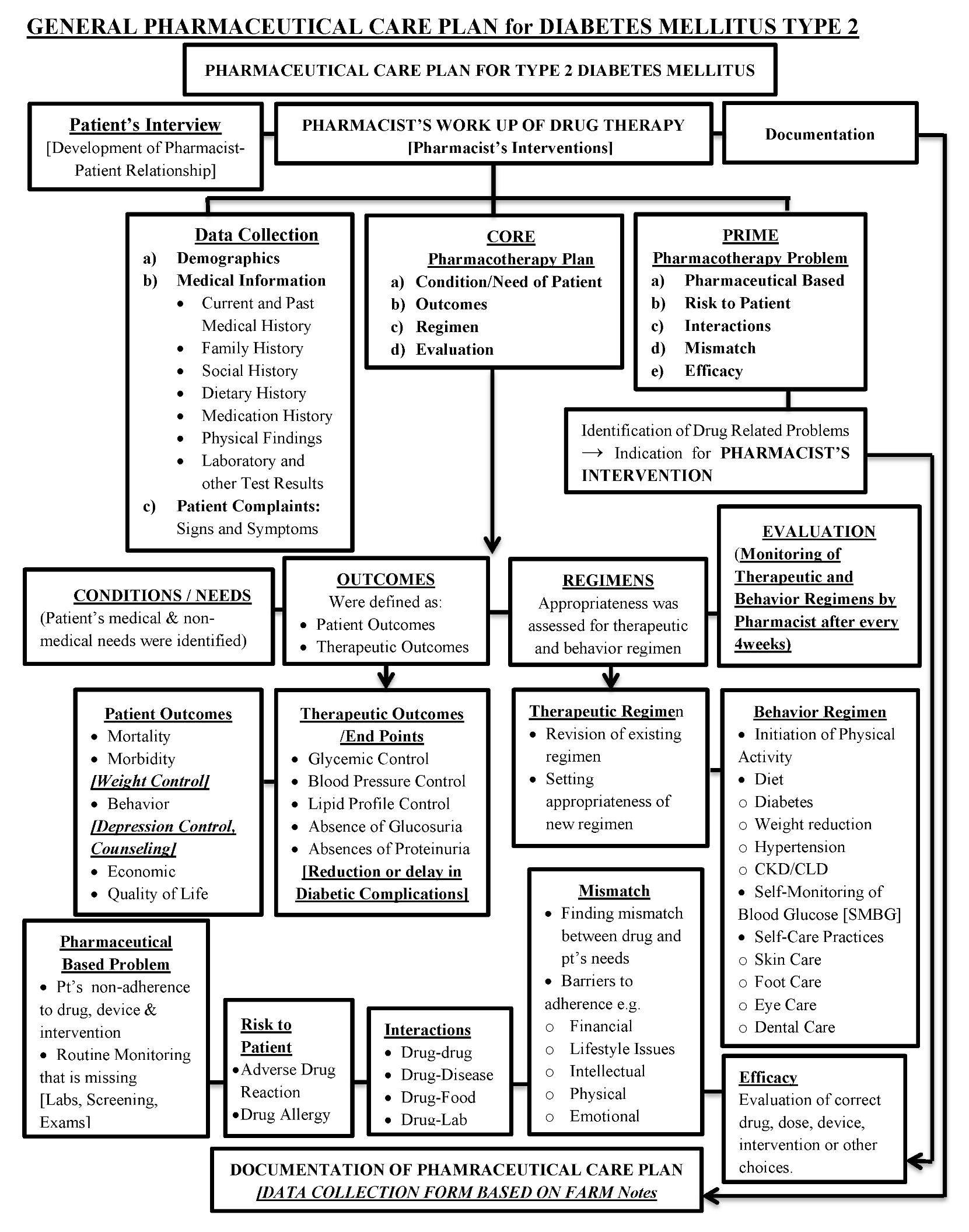


**Figure S2. Categorization of participants in intervention arm based on follow ups**

**
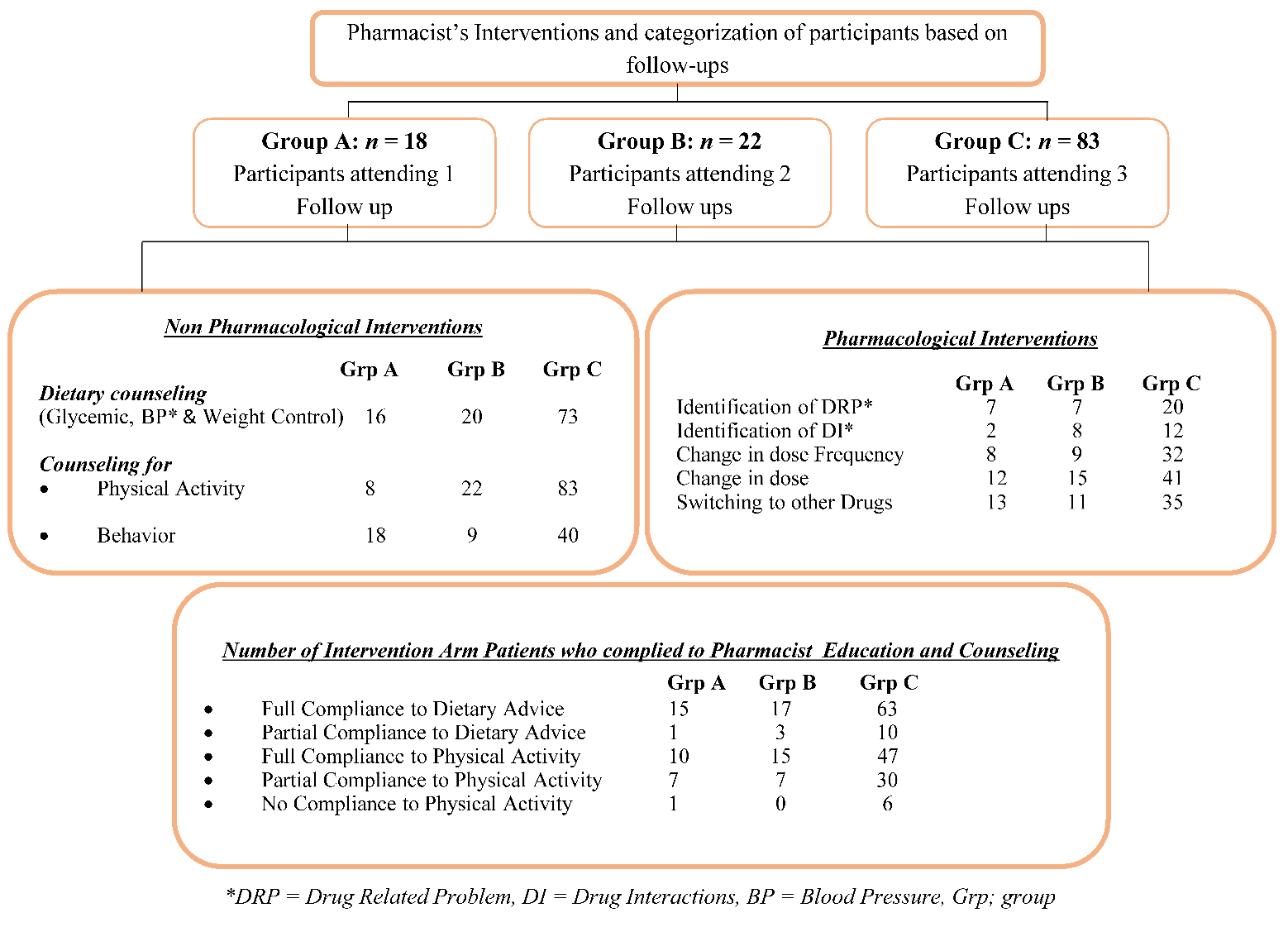
**

**Table S1: Patients Overall and Gender wise distribution of Baseline Demographics and Clinical Characteristics**

| **Demographics of the Participants**  **[*n* (**%**)]** | | | | |
| --- | --- | --- | --- | --- |
| **Parameters** | **Study Participants, *n*=244** | **Male, *n*=82** (33.6%) | **Female, *n*=162** (66.4%) | ***p*-values** |
| ***Marital Status***  Unmarried  Married  Widow | 10 (4.1)  216 (88.5)  18 (7.4) | 2 (2.4)  78 (95.1)  2 (2.4) | 8 (4.9)  138 (85.2)  16 (9.9) | 0.445 |
| ***Hypertension***  Yes  No | 189 (77.5)  55 (22.5) | 65 (34.4)  17 (30.9) | 124 (65.6)  38 (69.1) | 0.992 |
| ***Hepatosteatosis***  Yes  No | 144 (59)  100 (41) | 43 (29.8)  39 (39) | 101 (70.1)  61 (61) | 0.954 |
| ***Family History of Diabetes***  Yes  No | 176 (72.1)  68 (27.9) | 59 (33.5)  23 (33.8) | 117 (66.5)  45 (66.2) | 0.999 |
| ***Family History of Hypertension***  Yes  No | 146 (59.8)  98 (40.2) | 48 (32.9)  34 (34.7) | 98 (67.1)  64 (65.3) | 0.999 |
| **Overall Gender Wise Baseline Clinical Characteristics**  **[Mean ± SD]** | | | | |
| **Parameters** | **Study Participants, *n*=244** | **Male, *n*=82** | **Female, *n*=162** | ***p*-values** |
| ***Age (Yrs)*** | 50.4 **±** 9.2 | 49.5 **±** 9.3 | 50.8 **±** 9.2 | 0.30 |
| ***Duration of DM (Yrs)*** | 6.8 **±** 5.4 | 5.9 **±** 5.1 | 7.3 **±** 5.5.4 | 0.06 |
| ***Haemoglobin (mg/dL)*** | 13.2 **±** 1.1 | 14.2 **±** 1.1 | 12.7 **±** 0.6 | 0.0001** |
| ***Waist (cm)*** | 109.4 **±** 16.5 | 106.2 **±** 14.4 | 110.9 **±** 17.3 | 0.024* |
| ***BMI (Kg/m^2^)*** | 30.7 **±** 5.7 | 29.3 **±** 4.4 | 31.3 **±** 6.2 | 0.004* |
| ***Systolic BP (mmHg)*** | 138.9 **±** 19.3 | 138.4 **±** 15.2 | 139.3 **±** 21.1 | 0.75 |
| ***Diastolic BP (mmHg)*** | 89.7 **±** 11.3 | 89.7 **±** 8.8 | 89.7 **±** 12.5 | 0.99 |
| ***eABG (mg/dL)*** | 264.7 **±** 50.1 | 267.8 **±** 48.5 | 263.1 **±** 50.9 | 0.49 |
| ***HbA1c*** | 10.8 ± 1.7 | 10.9 **±** 1.7 | 10.8 **±** 1.8 | 0.49 |
| ***Cholesterol (mg/dL)*** | 226.7 **±** 53.6 | 238.3 **±** 61.3 | 220.7 **±** 48.4 | 0.026* |
| ***Triglycerides(mg/dL)*** | 231.8 ± 105.3 | 234.7 **±** 105.1 | 230.4 **±** 5.7 | 0.76 |
| ***HDL-C (mg/dL)*** | 48.3 **±** 15.6 | 47.9 **±** 15.1 | 48.4 **±** 15.9 | 0.82 |
| ***LDL-C (mg/dL)*** | 132 **±** 50.9 | 143.39 **±** 58.4 | 126.2 **±** 45.9 | 0.013* |
| ***VLDL-C (mg/dL)*** | 46.4 **±** 21.1 | 46.9 **±** 21.1 | 46.1 **±** 21.1 | 0.76 |
| ***Serum Creatinine (mg/dL)*** | 1 **±** 0.3 | 1.1 **±** 0.4 | 1.02 **±** 0.3 | 0.036* |
| ***Abbreviation: SD;*** *Standard Deviation,* ***DM;*** *Diabetes Mellitus,* ***BMI;*** *Body Mass Index,* ***eABG;*** *Estimated Average Blood Glucose;* ***HbA1c;*** *Glycated Haemoglobin,* ***HDL-C;*** *High Density Lipid-Cholesterol,* ***LDL-C;*** *Low Density Lipid-Cholesterol,* ***VLDL-C;*** *Very Low Density Lipid-Cholesterol,* ***M;*** *male,* ***F;*** *female*  ***p-values;*** **p < 0.05 – 0.002, ** p < 0.001 – 0.0001* | | | | |

**Table S2.** Percentage of Patients in HbA1c Reduction Quartiles

| **HbA1c Reduction Quartiles** | **Control Arm, n (%)** | | | **Intervention Arm, n (%)** | | |
| --- | --- | --- | --- | --- | --- | --- |
|  | **Follow Ups** | | | **Follow Ups** | | |
| From Baseline to follow ups | **1^st^ (***n*=121**)** | **2^nd^ (***n*=121**)** | **3^rd^ (***n*=52**)** | **1^st^ (***n*=123**)** | **2^nd^ (***n*=105**)** | **3^rd^ (***n*=82**)** |
| **< 1 %** | 38 (58.5) | 45 (53.6) | 17 (45.9) | 42 (34.4) | 16 (15.2) | 2 (2.4) |
| **1 – 1.9 %** | 21 (32.3) | 28 (33.3) | 18 (48.6) | 49 (40.2) | 22 (21) | 35 (42.2) |
| **2 – 2.9 %** | 4 (6.2) | 8 (9.5) | 1 (2.7) | 21 (17.2) | 32 (30.5) | - |
| **3 – 3.9 %** | 2 (3.1) | 2 (2.4) | 1 (2.7) | 8 (6.6) | 20 (19) | 24 (28.9) |
| **4 – 4.9 %** | - | 1 (1.2) | - | 1 (0.8) | 8 (7.6) | 9 (10.8) |
| **5 – 5.9 %** | - | - | - | 1 (0.8) | 5 (4.8) | 10 (12) |
| **6 – 6.9 %** | - | - | - | - | 1 (1) | 3 (3.6) |
| **≥ 7 %** | - | - | - | - | 1 (1) |  |
