# Supplementary material for: A randomized control trial of primary care-based management of type 2 diabetes by a pharmacist in Pakistan
Source: BMC Health Serv Res. 2019 Jun 24;19:409. doi: 10.1186/s12913-019-4274-z (PMC6591859; doi:10.1186/s12913-019-4274-z)
Supplement: Supplementary file 2 — Data collection form. (DOCX 76 kb) [file 12913_2019_4274_MOESM2_ESM.docx]

**Additional file 2. DATA COLLECTION FORM**

Name: S/O, D/O, W/O:

Marital Status: Pt. Code: Height (cm): _ Age/Gender: Occupation & Lifestyle:

Resident of:

Contact No.: Family History of DM: F/H HTN: _

Duration of DM (YRS): Current DM Therapy: INSULIN /OHA/DIET/NONE_ DIETARY Habits:

Co Morbidities: PRESENT COMPLAINT:

| **No.** | **Date** | **Weight (Kg)** | **Waist (cm)** | **BMI** | **BMR** | **R/Cal/d** | **Pharmacist Work Up / Notes** |
| --- | --- | --- | --- | --- | --- | --- | --- |
| 1 |  |  |  |  |  |  |  |
| 2 |  |  |  |  |  |  |  |
| 3 |  |  |  |  |  |  |  |
| 4 |  |  |  |  |  |  |  |
| 5 |  |  |  |  |  |  |  |
| 6 |  |  |  |  |  |  |  |
| 7 |  |  |  |  |  |  |  |
| 8 |  |  |  |  |  |  |  |

Process Measures:

| **No**  **.** | **Dat e** | **B.P**  **(mm/Hg)** | | **BSF**  **mg/d L** | **BSR**  **mg/d L** | **Hb A1c**  **(%)** | **LIPID PROFILE (mg/dL)** | | | | | | **S/Cr**  mg/dL  **0.5-1.2** | **eGFR**  **ml/min**  **/1.73m**  **2**  **AND**  **CKD –**  **Stage** |
| --- | --- | --- | --- | --- | --- | --- | --- | --- | --- | --- | --- | --- | --- | --- |
|  |  | **Sys** | **Dias** |  |  |  | **Total Lipids**  **450-**  **1000** | **Chol**  **≤200** | **TG 50-**  **150** | **HDL-C 45-65** | **LDL-C 0-150** | **VLDL**  **-C**  **0-30** |  |  |
| 1 |  |  |  |  |  |  |  |  |  |  |  |  |  |  |
| 2 |  |  |  |  |  |  |  |  |  |  |  |  |  |  |
| 3 |  |  |  |  |  |  |  |  |  |  |  |  |  |  |
| 4 |  |  |  |  |  |  |  |  |  |  |  |  |  |  |
| 5 |  |  |  |  |  |  |  |  |  |  |  |  |  |  |
| 6 |  |  |  |  |  |  |  |  |  |  |  |  |  |  |

Haematology Report

| **Date** | **Hb**  (g/dL)  ***[11.5-16]*** | **WBC TLC** (X103/µL) ***[4 – 11]*** | **ESR**  [mm/1st an hr]  ***[1 – 20]*** | **Total RBC** (X106/µL) ***[4.– 5.5]*** | **MCV**  (fL)  ***[76–96]*** | **HCT PVC** ( %)  ***[36–46]*** | **PLT**  (X103/µ L)  ***[150 –***  ***450]*** | **MCH**  (%)  ***[27 – 32]*** | **MCHC**  (%)  ***[30 – 35]*** | **N**  % | **L**  % | **M**  % | **E**  % | **Anti HCV** | **HBs Ag** | **SGPT**  **/ALT** (U/L) ***[5–34]*** |
| --- | --- | --- | --- | --- | --- | --- | --- | --- | --- | --- | --- | --- | --- | --- | --- | --- |
|  |  |  |  |  |  |  |  |  |  |  |  |  |  |  |  |  |
|  |  |  |  |  |  |  |  |  |  |  |  |  |  |  |  |  |

Urine Complete Examination Repor

| **Date** | **Col** | **Turb** | **Dep** | **S.G** | **pH** | **Gl** | **Prot** | **µ**  **Alb** | **Ket** | **Uro**  **bil** | **bil** | **bld** | **leuk** | **Pus**  **cell** | **Epith**  **cell** | **RBC** | **Cry** | **Bact** | **Muc** |
| --- | --- | --- | --- | --- | --- | --- | --- | --- | --- | --- | --- | --- | --- | --- | --- | --- | --- | --- | --- |
|  |  |  |  |  |  |  |  |  |  |  |  |  |  |  |  |  |  |  |  |
|  |  |  |  |  |  |  |  |  |  |  |  |  |  |  |  |  |  |  |  |

Any Other Lab Tests Performed during the study

| **Date** | **T3** | **T4** | **TSH** | **Foot Ex** | **Eye Ex** | **Tooth Ex** | **Skin Ex** |  |  |  |  |  |
| --- | --- | --- | --- | --- | --- | --- | --- | --- | --- | --- | --- | --- |
|  |  |  |  |  |  |  |  |  |  |  |  |  |
|  |  |  |  |  |  |  |  |  |  |  |  |  |

Non Pharmacological Interventions by Pharmacist (Patient Counseling) [ONLY FOR INTERVENTION ARM

PATIENTS]

| **No** | **Date** | **Time given** | **Disease (DSME)** | **SMBG** | **Hypo gly** | **Hyper gly** | **Diet/**  **Diet chart** | **Exercise** | **Med Adh** | **Eye care** | **Oral Care** | **Skin Care** | **Foot Care** | **Psychic Issues** | **Imm** | **Self Med** |
| --- | --- | --- | --- | --- | --- | --- | --- | --- | --- | --- | --- | --- | --- | --- | --- | --- |
| 1 |  |  |  |  |  |  |  |  |  |  |  |  |  |  |  |  |
| 2 |  |  |  |  |  |  |  |  |  |  |  |  |  |  |  |  |
| 3 |  |  |  |  |  |  |  |  |  |  |  |  |  |  |  |  |
| 4 |  |  |  |  |  |  |  |  |  |  |  |  |  |  |  |  |
| 5 |  |  |  |  |  |  |  |  |  |  |  |  |  |  |  |  |
| 6 |  |  |  |  |  |  |  |  |  |  |  |  |  |  |  |  |
| 7 |  |  |  |  |  |  |  |  |  |  |  |  |  |  |  |  |
| 8 |  |  |  |  |  |  |  |  |  |  |  |  |  |  |  |  |

Pharmacological Interventions by Pharmacist [ONLY FOR INTERVENTION ARM PATIENTS]:

**Prescription (Rx)**

| **No.** | **Date** | **Drug/Medicine** | **Dose** | **Change in Prescription** |
| --- | --- | --- | --- | --- |
|  |  |  |  |  |
|  |  |  |  |  |
|  |  |  |  |  |
|  |  |  |  |  |
|  |  |  |  |  |
|  |  |  |  |  |
