# Supplementary material for: A randomized control trial of primary care-based management of type 2 diabetes by a pharmacist in Pakistan
Source: BMC Health Serv Res. 2019 Jun 24;19:409. doi: 10.1186/s12913-019-4274-z (PMC6591859; doi:10.1186/s12913-019-4274-z)
Supplement: Supplementary file 3 — Patient’s consent form. (DOCX 291 kb) [file 12913_2019_4274_MOESM3_ESM.docx]

**Additional file 3:** Patient’s Consent Form

**IMPACT OF PHARMACIST INTERVENTION ON GLYCEMIC CONTROL IN TYPE 2 DIABETES MELLITUS (An Interventional Study)**

**Venue: ____________________________________________________________**

**Study Carried Out by:**

1. Zaida Javaid 2. Dr. Unaiza Imtiaz

Pharm. D (002516-A/001) MBBS (Pb.)

**Study Supervisor. Co Supervisor**

Dr. Hamid Saeed 1. Dr. Muhammad Imtiaz Khalid

Assistant Professor (TTS) MBBS (Pb) (15607-P)

Pharmacy Practice, Sr. Family Physician

University College of Pharmacy,

Punjab University, Lahore. 2. Zikria Saleem

Lecturer

University College of Pharmacy

Punjab University, Lahore

**Lab. Tests at:**

1. The Trust Laboratory, 2. Citilab & Research Center

Murad Medical Centre, 525 jinnah hospital chowk،

Main Shalamar Link Road, Lahore Maulana Shaukat Ali Road، Lahore

Supervised by: (for HbA1c & Lipid Profile)

Dr. Zafar Iqbal

MCPS, M. Phil Hematology Pt. Code: ___________________
